# Targeting CD300ld to normalize the tumor microenvironment: an emerging insight in cancer immunotherapy

**DOI:** 10.1002/mco2.607

**Published:** 2024-06-20

**Authors:** Lin‐Zhu Zhang, Xu He, Hai‐Dong Zhu

**Affiliations:** ^1^ Department of Radiology Center of Interventional Radiology and Vascular Surgery Medical School Nurturing Center of Jiangsu Province for State Laboratory of AI Imaging and Interventional Radiology (Southeast University) Zhongda Hospital Southeast University Nanjing China; ^2^ National Innovation Platform for Integration of Medical Engineering Education (NMEE) (Southeast University) Nanjing China; ^3^ Zhuhai Interventional Medical Center Zhuhai Precision Medical Center Zhuhai People's Hospital Zhuhai Hospital Affiliated with Jinan University Jinan University Zhuhai China

**Keywords:** immunotherapy, neutrophil, tumor

## Abstract

In a recent Nature elegant study, Wang et al. identified CD300ld, a novel functionally highly conserved tumor immunosuppressive receptor, highly expressed specifically on polymorphonuclear myeloid‐derived suppressor cells (PMN‐MDSCs), as well as a key receptor in the regulation of recruitment and immunosuppressive function of PMN‐MDSCs. Targeting CD300ld could remodel the tumor immune microenvironment, resulting in a broad‐spectrum anti‐tumor effect.

1

In a recent *Nature* elegant study, Wang et al. identified CD300ld as a tumor immunosuppressive receptor that is specifically highly expressed on polymorphonuclear myeloid‐derived suppressor cells (PMN‐MDSCs) with highly conserved functions.^1^ CD300ld can regulate the recruitment and immunosuppressive functions of PMN‐MDSCs. Targeting CD300ld can reshape the tumor immune microenvironment (TIM) and synergize with PD1 to produce stronger anti‐tumor effects (Figure 1).

**FIGURE 1:**
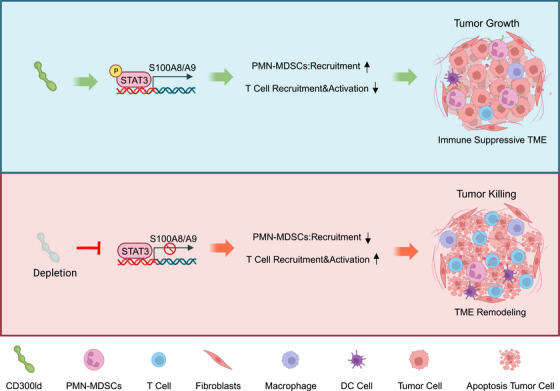
Inhibiting CD300ld could remodel the tumor's immune microenvironment. *CD300ld* deletion in polymorphonuclear myeloid‐derived suppressor cells (PMN‐MDSCs) exhibited significantly reduced levels of phosphorylated STAT3. This significant reduction in recruitment of STAT3 to the S100A8 and S100A9 promoters further modulates the recruitment of PMN‐MDSCs to tumors and inhibits T cell activation, thereby inhibiting tumor progression. Copyright permission^1^. [Created with BioRender.com (https://biorender.com)].

The presence of immunosuppressive myeloid cells in TIM impedes the effectiveness of tumor treatment and poses a significant challenge to current immunotherapy. Extended exposure of myeloid progenitor cells to growth factors secreted by tumors as well as inflammatory mediators stimulates abnormal granulopoiesis, remarkably increasing the release of neutrophils from the bone marrow. In advanced cancers, functionally disturbed neutrophils display high levels of plasticity and potent immunosuppressive activity, generally implied as PMN‐MDSCs.^2^ There are various mechanisms for PMN‐MDSCs to facilitate tumor progression and impede anti‐cancer immune responses. PMN‐MDSCs express multiple mediators that regulate T cell function leading to anti‐tumor immune disorders. In addition to affecting immune responses, reactive oxygen species secreted by PMN‐MDSCs cause DNA damage and genetic instability in tumors. Targeted inhibition of the function of PMN‐MDSCs in the tumor microenvironment has emerged as a promising cancer therapeutic strategy, especially when combined with immune checkpoint inhibitors that show superior anti‐tumor properties.^3^ It is worth noting that off‐target side effects and compensatory myelopoiesis due to the regulation of physiological homeostasis should also be kept in mind when developing therapeutic agents targeting PMN‐MDSCs. So far, only a few phenotypic and genetic markers can be used to distinguish PMN‐MDSCs from normal neutrophils. Discovering specific targets against PMN‐MDSCs to modulate TIM is the current focus and challenge of immunotherapy.

Myeloid cells infiltrating into tumors are deeply involved in tumor progression, and membrane proteins have adequate targeting and druggability. Wang et al. used Cas9 mice to conduct in vivo CRISPR‐Cas9 screening and found that the membrane protein CD300ld was significantly deleted in PMN‐MDSCs. CD300ld is a single transmembrane protein of the CD300 protein family. The CD300 family of proteins on myeloid cell membranes has the dual function of stimulating and inhibiting cellular function.^4^ Among them, when CD300lf and CD300ld form paired receptors, they may recognize the same ligand and maintain cellular balance in opposite ways.^4^ Previous reports considered it a potential target of mouse norovirus‐invading cells that may be involved in the activation process of neutrophils. However, the function of CD300ld in the occurrence of tumors has not yet been studied. Wang et al. found that CD300ld is mainly highly expressed in neutrophils as well as upregulated in PMN‐MDSCs generated after tumor‐bearing, which indicates that CD300ld may be involved in tumor progression.^1^ *CD300ld* knockout mice confirmed that *CD300ld* deletion not only significantly inhibited the development of various secondary and primary tumor models but also had no significant impact on the normal development of the mouse body and immune system. The tumors in *CD300ld* gene‐deficient mice had less accumulation of PMN‐MDSCs, and the number of immune effector cells, such as CD4^+^ and CD8^+^ T cells, and natural killer cells, was significantly increased. Therefore, CD300ld was identified as a key functional receptor for PMN‐MDSCs to promote tumor development.

The CD300 family members are usually expressed on leukocyte membranes, and CD300a and CD300c are widely expressed in most leukocyte populations. Other members of the CD300 molecular family show bone marrow‐restricted expression. Among them, CD300d was found on the endoplasmic reticulum membrane and cannot fuse with the cell membrane unless combined with a molecular chaperone. The CD300 family molecules play a role in regulating immune responses through paired triggering and inhibitory effects. Wang et al. found that the expression of CD300ld is limited to myeloid cells, with low or no expression in other myeloid cells or lymphoid cells. Transcriptome sequencing identified S100A8/A9 as a key effector molecule downstream of CD300ld. This finding compensates for the limitations of Kim et al.’s study on how ferroptosis mediates the downstream pathways of immunosuppression exerted by PMN‐MDSCs.^5^ Subsequently, it was found that significant anti‐tumor effects still existed when blocking CD300ld after tumor formation. CD300ld was competitively blocked by the injection of CD300ld extracellular domain protein, presenting similarly to *CD300ld* deletion, and showed meaningful anti‐tumor cooperation with anti‐PD1, implying the potential of CD300ld to be a therapeutic target.

The TCGA database and patient samples of several different tumor types were further analyzed. The results show that CD300ld is also highly expressed in human PMN‐MDSCs and is closely related to neutrophil infiltration and patient prognosis. Competitive inhibition of humanized *CD300ld* mice also showed significant anti‐tumor effects. In addition, studies have reported that the extracellular domain of mouse CD300ld is highly similar to CD300lf. Whether the same therapeutic effect as CD300ld can be achieved by blocking CD300lf remains to be further verified.

In this study, CD300ld, a membrane protein molecule specifically highly expressed in neutrophils or PMN‐MDSC, was screened and identified, and this molecule is deeply involved in tumor progression. It provides vital clinical significance and a basis for drug development. CD300ld inhibitors represent a promising yet challenging area for future research. The development of CD300ld inhibitors may greatly compensate for other tumors that are insensitive or ineffective to programmed death‐ligand 1, immunotherapy. It is unknown whether immunotherapy targeting CD300ld can be used clinically with minimal risk of side effects. Second, the endogenous ligand of CD300ld has not yet been characterized. Lastly, whether an upstream signal initiating the CD300ld pathway exists needs to be further studied.

Currently, in addition to reshaping TIM through immune checkpoint inhibitor therapy, treatments such as adoptive immune cell therapy, T cell activators, and multispecific antibodies may be applied to patients in the near future. To further enhance tumor treatment efficacy, targeting PMN‐MDSC may be the key to the new generation of immunotherapy. To understand the functional complexity of PMN‐MDSC and its interaction with various cells within TIM and their possible reprogramming, future studies should focus on intertissue‐resident stromal cells and immune cells as well as those outside the tumor (bone marrow and spleen)‐derived PMN‐MDSCs. In real‐world solid cancer treatment, radical surgical resection of tumor tissue remains the treatment of choice. However, a large number of patients experience tumor recurrence after surgery. Paradoxically, however, tissue damage caused during surgery may inhibit innate and adaptive immunity and accelerate the spread of PMN‐MDSCs to promote tumor cell metastasis and proliferation of residual cancer cells.

In summary, this study discovered the key functional receptor CD300ld on the surface of PMN‐MDSCs, which plays an important role in tumor immunosuppression. CD300ld simultaneously regulates the recruitment of PMN‐MDSCs and inhibits T‐cell infiltration through the STAT3‐S100A8/A9 axis. Blocking CD300ld may remodel the immune microenvironment from an immunosuppressive state to an activated state by reducing the recruitment of PMN‐MDSCs and decreasing their immunosuppressive function, opening the door to personalized targeted therapy.

## AUTHOR CONTRIBUTIONS

**Lin‐Zhu Zhang**: writing–original draft; writing–review; and editing (supporting). **Xu He**: project administration (leading); writing–review and editing (supporting). **Hai‐Dong Zhu**: conceptualization (leading); funding acquisition (leading); writing–review and editing (leading). All authors have read and approved the article.

## CONFLICT OF INTEREST STATEMENT

The authors declare no conflict of interest.

## ETHICS STATEMENT

Not applicable.

## Data Availability

Not applicable.
